# Quantitative Lipid Profiling Reveals Major Differences between Liver Organoids with Normal Pi*M and Deficient Pi*Z Variants of Alpha-1-antitrypsin

**DOI:** 10.3390/ijms241512472

**Published:** 2023-08-05

**Authors:** Sara Pérez-Luz, Jaanam Lalchandani, Nerea Matamala, Maria Jose Barrero, Sara Gil-Martín, Sheila Ramos-Del Saz, Sarai Varona, Sara Monzón, Isabel Cuesta, Iago Justo, Alberto Marcacuzco, Loreto Hierro, Cristina Garfia, Gema Gomez-Mariano, Sabina Janciauskiene, Beatriz Martínez-Delgado

**Affiliations:** 1 Molecular Genetics and Genetic Diagnostic Units, Institute of Rare Diseases Research (IIER), Spanish National Institute of Health Carlos III (ISCIII), 28220 Madrid, Spain; sara.perez@isciii.es (S.P.-L.); j.lalchandani@isciii.es (J.L.); nmatamala@isciii.es (N.M.); sara.gil@externos.isciiii.es (S.G.-M.); sheila.ramos@isciii.es (S.R.-D.S.); ggomezm@isciii.es (G.G.-M.); 2 Models and Mechanisms Unit, Institute of Rare Diseases Research (IIER), Spanish National Institute of Health Carlos III (ISCIII), 28220 Madrid, Spain; mj.barrero@isciii.es; 3 Centro de Investigación Biomédica en Red de Enfermedades Raras, CIBERER U758, 28029 Madrid, Spain; 4 Bioinformatics Unit, Institute of Health Carlos III (ISCIII), 28220 Madrid, Spain; s.varona@isciii.es (S.V.); smonzon@isciii.es (S.M.); isabel.cuesta@isciii.es (I.C.); 5 General and Digestive Surgery Department, Hospital 12 de Octubre, 28041 Madrid, Spain; iagojusto@hotmail.com (I.J.); alejandro_mar@icloud.com (A.M.); 6 Paediatric Hepatology Service, Research Institute of University Hospital La Paz, (IdiPAZ), 28046 Madrid, Spain; marial.hierro@salud.madrid.org; 7 Digestive Department, Hospital 12 de Octubre, 28041 Madrid, Spain; cgarfia@hotmail.com; 8 Department of Respiratory Medicine, Member of the German Center for Lung Research (DZL), Biomedical Research in Endstage and Obstructive Lung Disease Hannover (BREATH), Hannover Medical School, 30625 Hannover, Germany; janciauskiene.sabina@mh-hannover.de

**Keywords:** alpha-1 antitrypsin deficiency (AATD), alpha-1 antitrypsin (AAT), liver organoids, AAT aggregates, lipid accumulation

## Abstract

Different mutations in the *SERPINA1* gene result in alpha-1 antitrypsin (AAT) deficiency and in an increased risk for the development of liver diseases. More than 90% of severe deficiency patients are homozygous for Z (Glu342Lys) mutation. This mutation causes Z-AAT polymerization and intrahepatic accumulation which can result in hepatic alterations leading to steatosis, fibrosis, cirrhosis, and/or hepatocarcinoma. We aimed to investigate lipid status in hepatocytes carrying Z and normal M alleles of the *SERPINA1* gene. Hepatic organoids were developed to investigate lipid alterations. Lipid accumulation in HepG2 cells overexpressing Z-AAT, as well as in patient-derived hepatic organoids from Pi*MZ and Pi*ZZ individuals, was evaluated by Oil-Red staining in comparison to HepG2 cells expressing M-AAT and liver organoids from Pi*MM controls. Furthermore, mass spectrometry-based lipidomics analysis and transcriptomic profiling were assessed in Pi*MZ and Pi*ZZ organoids. HepG2 cells expressing Z-AAT and liver organoids from Pi*MZ and Pi*ZZ patients showed intracellular accumulation of AAT and high numbers of lipid droplets. These latter paralleled with augmented intrahepatic lipids, and in particular altered proportion of triglycerides, cholesterol esters, and cardiolipins. According to transcriptomic analysis, Pi*ZZ organoids possess many alterations in genes and cellular processes of lipid metabolism with a specific impact on the endoplasmic reticulum, mitochondria, and peroxisome dysfunction. Our data reveal a relationship between intrahepatic accumulation of Z-AAT and alterations in lipid homeostasis, which implies that liver organoids provide an excellent model to study liver diseases related to the mutation of the *SERPINA1* gene.

## 1. Introduction

Alpha-1 antitrypsin deficiency (AATD) is a rare inherited disorder (ORPHA 60) most prevalent among Caucasians (1:2000–5000 people). AATD is characterized by low levels of circulating alpha-1 antitrypsin (AAT), it is a risk factor for developing lung and/or liver diseases, as well as neutrophilic panniculitis or systemic vasculitis in some cases [1]. AATD is a monogenic condition caused by mutations in the *SERPINA1* gene [2] encoding AAT, an acute phase glycoprotein and a major inhibitor of neutrophil elastase primarily synthetized in hepatocytes (by about 80%) and then released into the bloodstream, it also expressed to a lesser extent by monocytes and macrophages [3,4]. In addition, AAT is regarded as a multifunctional immunomodulatory protein that inhibits apoptosis and binds and neutralizes activities of cytokines and oxidants, among others [5,6].

The *SERPINA1* gene is localized in the long arm of chromosome 14, is composed of 7 exons, and has more than 150 allelic variants [7]. Some of the variants seem to have no clinical relevance and are classified as “normal” or as a typical “M” variant. Clinically relevant, so-called deficient, variants result from point mutations or small deletions in the *SERPINA1* gene which lead to the low or undetectable levels of circulating AAT protein. The S allele (Glu206Val) originating from a point mutation in exon 3 and the Z allele (Glu342Lys) from a point mutation in exon 5 are the most common. In fact, about 96% of clinically recognized AATD cases carry the Z allele in homozygosity and only about 4–5% are heterozygous (Pi*SZ or Pi*MZ) or contain other rare alleles [2,8]. The Z allele is the most clinically relevant [7]. The Z mutation alters the normal folding of the AAT protein and triggers its polymerization. Therefore, hepatic manifestations are related to “gain of function mechanisms” due to the intrahepatic accumulation and cytotoxicity of Z-AAT polymers [9]. By contrast, lung pathologies are linked to “loss of function mechanisms” due to the low circulating monomeric and high polymeric Z-AAT having insufficient antiprotease/anti-inflammatory activities [3], notwithstanding that other epigenetic factors can also contribute [10,11].

Recent studies described lipid alterations in Pi*ZZ patients with liver diseases. Specifically, serum levels of triglyceride and cholesterol levels were found to be lower in AATD in comparison to non-AATD patients [12,13]. Transgenic mice expressing the human Z allele also showed alterations in hepatic lipid metabolism, namely increased levels of hepatic triglycerides and cholesterol [14], and high numbers of lipid droplets [12]. Lipids are key cellular components necessary for maintaining the integrity of the cellular membranes and energy homeostasis, although they may also contribute to pathologies [15]. Specifically, the formation of intracellular lipid droplets (lipid storage) can trigger pathological mechanisms [16].

Hepatic organoids are new models that reproduce the main characteristics of the liver and are useful for the modeling of hepatic disease or drug screening [17,18]. Liver organoids offer possibilities for deepening the understanding of the molecular mechanisms underlying Z-AAT polymerization and the related consequences of AATD, and for testing new therapeutic strategies. Herein, we used hepatic organoids derived from liver biopsies obtained from a Pi*MZ and a Pi*ZZ patient (MZ-ORG and ZZ-ORG) and from a Pi*MM, non-deficient individual (MM-ORG), with the aim of checking whether liver organoid cultures also recapitulate the hepatic steatosis found in patients with AATD and investigating the molecular alterations related to such lipid deposits leading to liver damage. Experimental data based on Z-AAT expressing HepG2 cells and AATD patient-derived organoids confirmed a link between AAT polymer accumulation and changes in hepatic lipid metabolism.

## 2. Results

### 2.1. Intracellular Accumulation of Z-AAT Polymers Parallel with Increased Lipid Content

**Figure 1:**
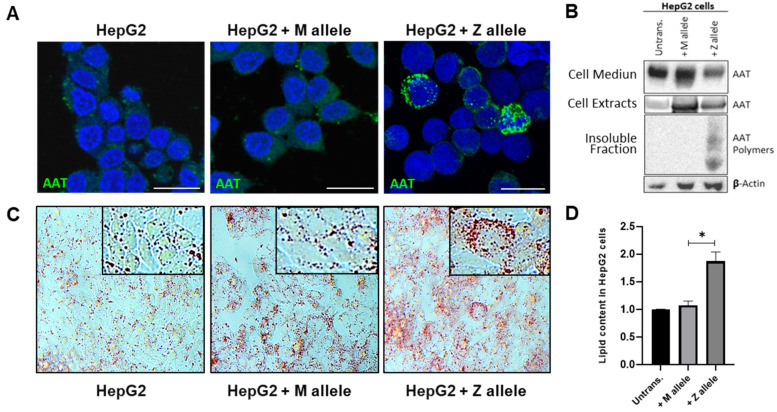
Z-AAT protein expression in HepG2 cells and lipid accumulation in hepatocytes. (**A**) Representative immunofluorescence photomicrographs of HepG2 cell lines (not transfected and expressing M or Z alleles) labelled in green with AAT and in DAPI (blue) for nuclei; scale bar represents 25 µm. (**B**) Representative Western blotting showing AAT protein levels after transfection of HepG2 cells. (**C**,**D**) Oil-Red-O staining and quantification of lipids in transfected cells compared to control cells; images were taken with a 20× magnification. The values are expressed as mean ± SEM in relative percentage of the control (* *p* ≤ 0.05).

We first used HepG2 cells expressing Z- or M-AAT to check for intracellular AAT and lipid accumulation. As shown in Figure 1, HepG2 cells transfected with either M or Z alleles expressed higher amounts of intracellular protein in comparison to transfected cells when immunohistochemistry was performed to detect total AAT protein (Figure 1A). As expected, cell lines containing M-AAT plasmids expressed a high amount of monomeric AAT protein, whereas cells transfected with Z-AAT plasmid expressed Z-AAT polymers which retained in the insoluble cell fraction (Figure 1B). Remarkably, HepG2 cells expressing Z-AAT polymers, but not M-AAT monomers, contained a lot of intracellular lipid droplets (LDs) stained with Oil-Red O (Figure 1C, D).

**Figure 2:**
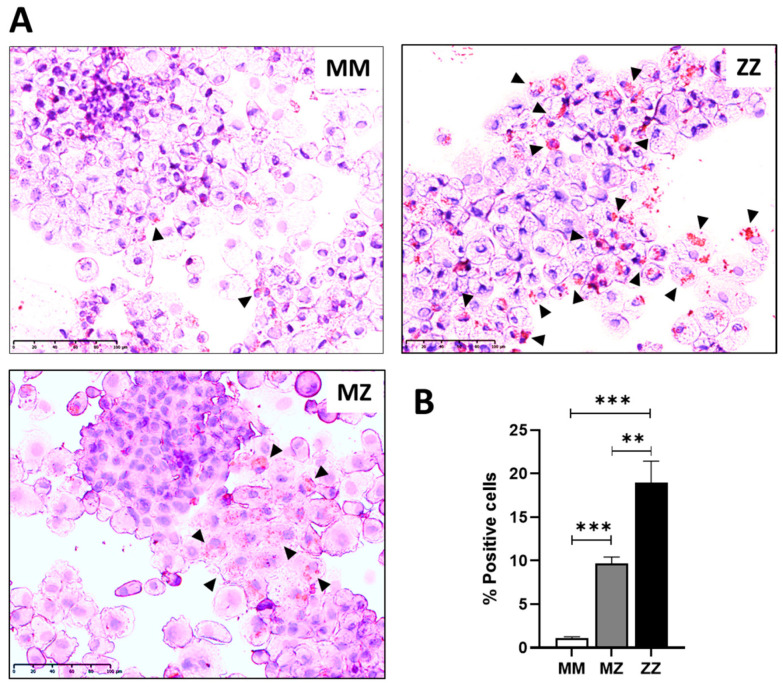
Oil-Red-O staining in MM, MZ, and ZZ organoids. (**A**) Representative images of ORO staining in organoids derived from different AAT genotypes. Arrowheads point to cells showing positive lipid staining. (**B**) Number of positive cells for ORO staining. At least 10 fields of three different cytospin preparations were used for quantification; scale bar represents 100 µm (** *p* ≤ 0.01, *** *p* ≤ 0.005).

To further investigate if there is a link between intracellular Z-AAT polymers and lipid accumulation, we performed Oil-Red O staining in liver organoids (Figure 2). When compared to MM-ORG, MZ-, but especially ZZ-ORG, had a high number of cells containing LDs which paralleled with Z-AAT polymer accumulation (Figure 2A, B).

### 2.2. Differences in Lipidomic Profiles between MM-, MZ-, and ZZ-ORGs

**Figure 3:**
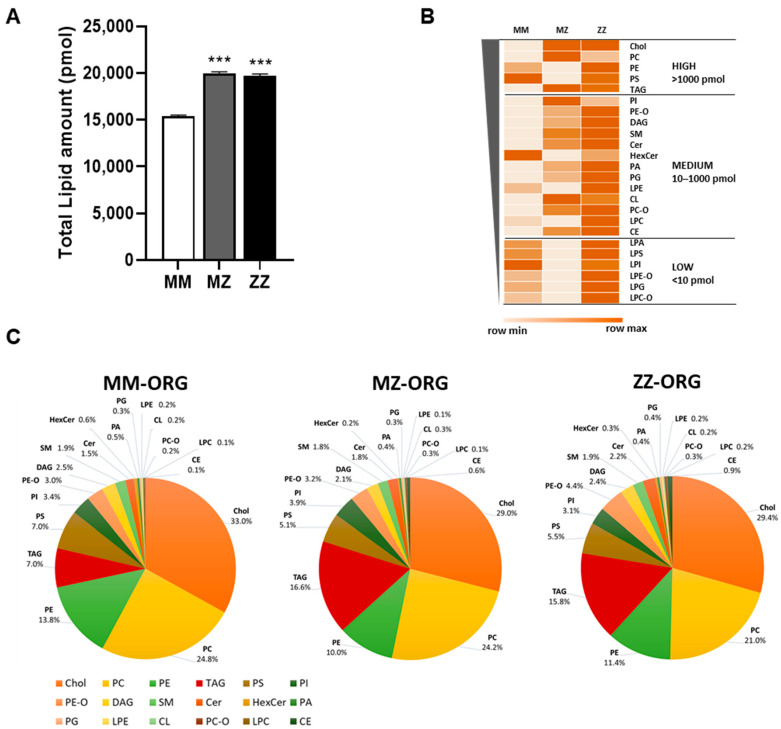
Lipid content in AATD-derived organoids. (**A**) Total amount of lipids (pmol) in MZ and ZZ organoids compared to MM ones; data represent the mean of three different replicates analyzed (*** *p* ≤ 0.005). (**B**) Heat-map showing the mean amount of each lipid in the MM, MZ, and ZZ organoids. Lipid species are ordered from the most abundant in MM organoids at the top to the lowest at the bottom. Groups separating the most highly abundant lipids (>1000 pmol), intermediate level (10–1000 pmol), and low abundant (>10 pmol) are represented. (**C**) Diagrams showing the percentages, relative to the total amount, of each lipidic species in the MM-, MZ-, and ZZ-ORG. The lipid distribution in MZ- and ZZ-ORG show more similar profiles compared to MM-ORG.

Mass spectrometry and quantitative lipidomic analyses were used to determine membrane and intracellular lipids. As shown in Figure 3A, the total content of lipids was significantly higher in MZ- and ZZ-ORG than in MM-ORG. A more detailed analysis revealed specific lipid species being substantially high in MZ- but even higher in ZZ-ORG compared to in MM-ORG (Table 1). As illustrated in Figure 3B, ZZ-ORG showed the largest number of significantly altered lipid species while MZ-ORG had an intermediate profile. Among the most abundant intracellular lipids (those with >1000 pmol), there were triglycerides (TAG), cholesterol (Chol), and phosphatidylcholine (PC) which were clearly higher in MZ- and ZZ-ORG than in MM-ORG. C lipids of intermediate abundance (10–1000 pmol), ceramides (CERs), cardiolipins (CLs), phosphatidylcholine (-ether) (PC-O), phosphatidylethanolamine (-ether) (PE-O), and cholesterol esters (CEs) were found to be higher in MZ- and ZZ-ORG relative to MM-ORG. Lipids with lower abundance (<10 pmol) included different lysophospholipids, many of which were not detected in MZ-ORG but were high in ZZ-ORG (Figure 3B).

### 2.3. Distribution of Specific Lipids within ZZ-, MZ-, and MM-ORG Relative to the Total Lipid Content

Examining the percentages of the specific lipid classes within the total lipid content, we found that MZ- and ZZ-ORG had more similar lipid profiles in comparison to MM-ORG (Figure 3C). Among the dominant lipid species in MZ- and ZZ-ORG were TAG, PE-O, CER, CL, PC-O, and CE (Table 2). Specifically, TAG constituted 7% of total lipids in MM-ORG but was significantly increased by nearly 16% in MZ- and ZZ-ORG (*p* < 0.0001). Similarly, CE represented less than 0.1% of lipids in MM-ORG but was increased by 0.6–0.8% in MZ- and ZZ-ORG (*p* < 0.0001). By contrast, the proportion of other lipid species, such as PC, was significantly lower in ZZ-ORG relative to MZ-ORG (*p* = 0.0066) or MM-ORG (*p* = 0.0028) (Figure 3C, Table 2).

### 2.4. Functional Annotation Analysis of Differentially Expressed Genes (DEGs) between ZZ- and MM-ORG

To characterize MM- and ZZ-ORG in more detail, we performed a transcriptome analysis. When comparing ZZ- and MM-ORG, we found 633 DEGs, among which 345 were up-regulated and 288 down-regulated in ZZ- versus MM-ORG. Functional annotation analysis of DEGs based on enrichment of Gene Ontology (GO) terms and KEGG pathways revealed five gene clusters related to different functions (Table 3). Cluster 1 included genes of actin binding (False Discovery Rate, FDR: 8.25 × 10^−7^), cytoskeleton organization (FDR: 0.0017), and vesicles (FDR: 0.039), such as WAS/WASL, interacting protein family member 3 (*WIPF3*), FH2 domain containing 1 (*FHDC1*), actin related protein 3C (*ACTR3C*), and transferrin receptor (*TFRC*). On the other hand, the cluster 2 included genes associated with metabolic process (FDR: 1.45 × 10^−9^), regulation of gene expression (FDR: 3.64 × 10^−9^), response to stress (FDR: 4.68 × 10^−6^), and inflammatory response (FDR: 0.001). Specifically, this cluster included genes encoding enzymes like phospholipase C beta 1 (*PLCB1*), argininosuccinate synthase 1 (*ASS1*), insulin like growth factor binding protein 5 (*IGFBP5*) or cytochrome p450 oxidoreductase (*POR*), and a number of transcription factors, such as GATA binding protein 3 (*GATA3*), Jun proto-oncogene, AP-1 transcription factor subunit (*JUN*), activating transcription factor 3 (*ATF3*), MAF bZIP transcription factor (*MAF*), MYCN proto-oncogene, and bHLH transcription factor (*MYCN*) which may regulate complex processes related to cellular stress response and inflammatory response. Furthermore, cluster 2 included genes encoding alpha-2-macroglobulin (*A2M*) and serpin family members (*SERPINE1*, *SERPINF1*), which are involved in the regulation of proteolysis. Cluster 3 contained genes related to liver metabolism such as drug metabolism (FDR: 4.22 × 10^−6^), fatty acid metabolic process (FDR:0.04), and bile secretion (FDR:0.0025). Among other genes were *CD36*, acyl-CoA thioesterase 1 and 2 (*ACOT1*, *ACOT2*) carnitine O-octanoyltransferase (*CROT*), cytochrome P450 family members (*CYP2A6*, *CYP1B1*), and some UDP glucuronosyltransferase (*UGT2B10*, *UGT2B28*, *UGT1A4*, *UGT1A5*). Cluster 4 formed genes related to the intrinsic components of plasma membrane (FDR: 0.02) and cell junctions (FDR:0.001), whereas a cluster 5 included genes of the organelle and endomembrane system (FDR: 0.002), Golgi apparatus (FDR:0.002), and cation channel complexes. In this latter cluster were Mannosidase alpha class 1A member 1 (*MAN1A1*) and genes involved in ER to Golgi vesicle-mediated transport, such as Bet1 Golgi vesicular membrane trafficking protein (*BET1*), KDEL endoplasmic reticulum protein retention receptor 2 (*KDELR2*), or YKT6 V-SNARE homolog (*YKT6*), as well as different polypeptide N-acetylglucosaminyltransferases (*GALNT12*, *GALNT5*, *GALNT6*).

### 2.5. Gene Set Enrichment Analysis (GSEA) in ZZ-ORG Versus MM-ORG

**Figure 4:**
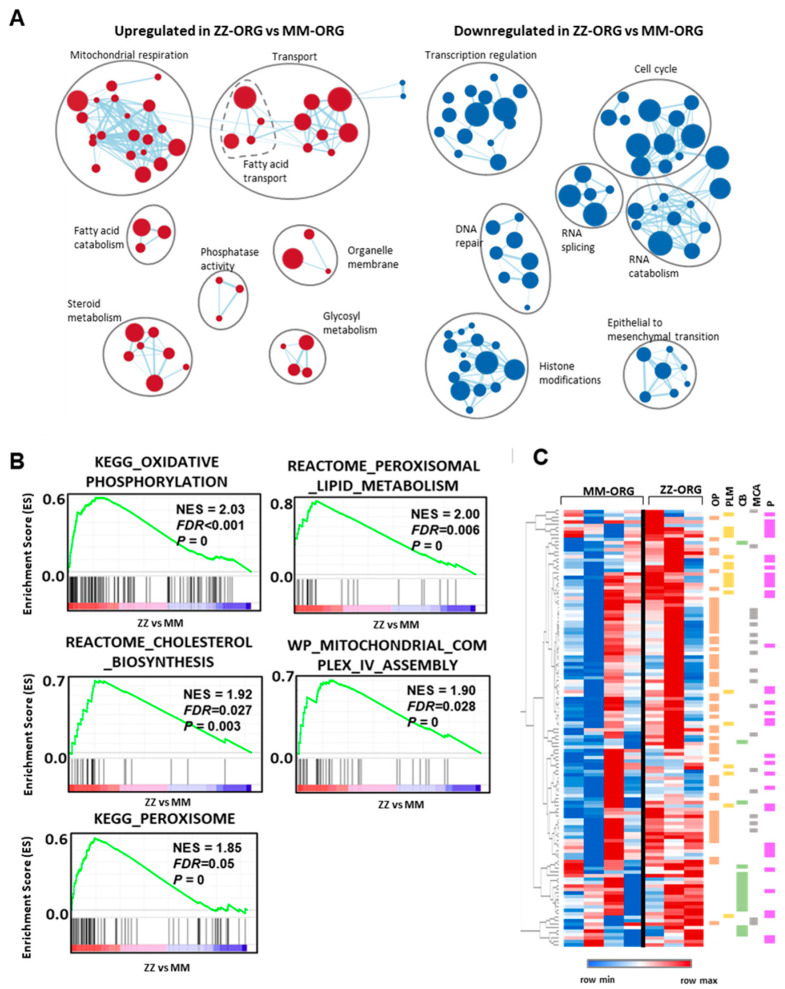
GSEA analysis of ZZ-ORG vs. MM-ORG differential gene expression. (**A**) Clusters of GO gene sets differentially enriched in ZZ-ORG vs. MM-ORG at *p*-value < 0.01 according to preranked GSEA analysis. Node size is proportional to the number of genes identified in each gene set. The light blue edges indicate gene overlap between gene sets. (**B**) Graphs of positive enrichment scores (ESs) found in preranked GSEA analysis in ZZ-ORG compared to MM-ORG. (**C**) Unsupervised hierarchical cluster heatmap of mRNA levels of genes belonging to the leading edge of pathways depicted in B. Lower expression of genes is represented in blue and higher expression in red. Genes belonging to the pathways of oxidative phosphorylation (OP) are in orange, peroxisomal lipid metabolism (PLM) in green, cholesterol biosynthesis (CB) in yellow, mitochondrial complex IV assembly (MCA) in grey, and peroxisome (P) in pink are indicated.

We used GSEA to interpret gene expression data and find differentially enriched functional terms in ZZ-ORG comparing to MM-ORG (Figure 4A). The top upregulated genes in ZZ-ORG revealed significant GO terms such as cellular transport, fatty acid transport, mitochondrial respiration, fatty acid catabolism, steroid metabolism, glycosyl metabolism, phosphatase activity, and organelle membranes. On the other hand, highly downregulated genes appear grouped in GO terms as cell cycle, transcription regulation, RNA splicing, RNA catabolism, DNA repair, histone modification, and epithelial to mesenchymal transition (Figure 4A).

Furthermore, different established pathways (KEGG, Reactome, WP) were also found significantly upregulated in ZZ-ORG relative to MM-ORG, revealing 126 genes involved in oxidative phosphorylation (FDR < 0.001), mitochondrial complex IV assembly (FDR = 0.028), cholesterol biosynthesis (FDR = 0.027), peroxisome (FDR = 0.05), and peroxisomal lipid metabolism (FDR = 0.006) (Figure 4B). The unsupervised clustering of the genes in the leading edge of the gene sets (Figure 4B) showed that genes belonging to these pathways are coordinately overrepresented in ZZ-ORGs (Figure 4C).

### 2.6. Expression Levels of Specific DEGs Related to Lipid Transport and Metabolism

**Figure 5:**
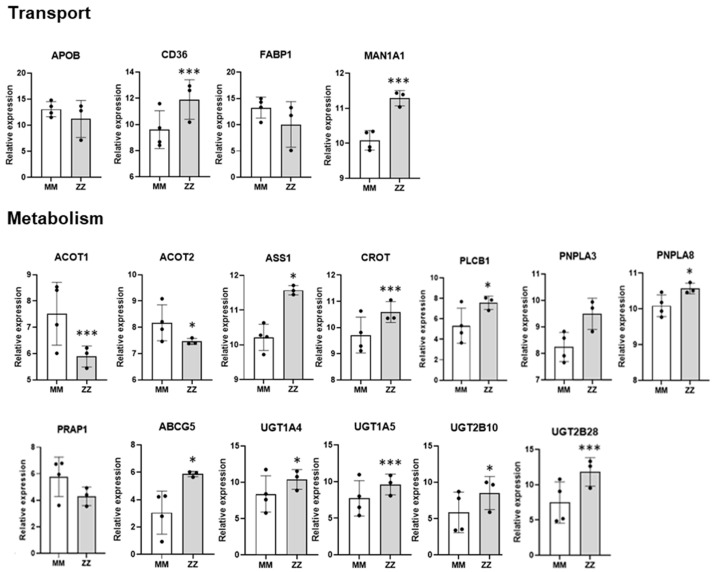
Relative expression of deregulated genes in the RNASeq analysis. The main differentially expressed genes implicated in lipid homeostasis are represented, grouped according to those involved in lipid transport (*APOB*, *CD36*, *FABP1* and *MAN1A1*); or different processes of lipid metabolism (*ACOT1*, *ACOT2*, *ASS1*, *CROT*, *PLCB1*, *PNPLA3*, *PNPLA8*, *PRAP1*, *ABGC5*, *UGT1A4*, *UGT1A5*, *UGT2B10*, *UGT2B28*). Data represent the mean ± SEM from three different replicates. * *p* < 0.05; *** *p* < 0.001.

Focusing on different aspects of lipid homeostasis, we found several DEGs related to lipid transport and metabolism (Figure 5). In comparison with MM-ORG, ZZ-ORG showed lower expression of Fatty Acid Binding Protein 1, *FABP1* (intracellular lipid transporter), and Proline Rich Acidic Protein 1, *PRAP1* (promoter of lipoprotein assembly and secretion). Moreover, lipid metabolism genes, such as *ACOT1* and *ACOT2,* involved in the regulation of fatty acid oxidation were also downregulated in ZZ-ORG. On the other hand, ZZ-ORG showed higher expression of *CD36* (a long-chain fatty acids transporter), *CROT* (a gene involved in peroxisomal lipid metabolism and fatty acid beta-oxidation), *PNPLA3* (a TAG hydrolase), *PNPLN8* (which catalyses the cleavage of fatty acids from membrane phospholipids), *ASS1* (an enzyme of the urea cycle), and *ABCG5* (involved in sterol excretion by the liver into bile).

## 3. Discussion

Homozygous Pi*ZZ AATD is a genetic condition caused by the incorrect folding of the Z-AAT protein, which through different molecular mechanisms can affect the liver and/or lungs [1,3]. Although most heterozygotes Pi*MZ individuals remain clinically healthy, the Z-allele is still considered a genetic modifier and a risk factor for cirrhosis in alcoholic and non-alcoholic fatty liver diseases [19]. The pathophysiological mechanisms implicated in Pi*Z AATD-related liver diseases are still incompletely understood.

Human organoids, as a novel model system, are very useful in studying organ health and diseases, as well as the effects of treatments [20,21]. Three-dimensional liver organoids are becoming more widely used for developmental and chronic disease modelling because they represent human liver cells more accurately than in vitro cell cultures lacking 3D tissue organization or in vivo animal models which are expensive, difficult to generate, and cannot completely recapitulate the underlying mechanisms of human diseases. For example, previous work from our group and others shows that liver organoids derived from Pi*ZZ AATD patients mimic the main liver features, are, namely, positive for PAS (Periodic acid-Schiff) diastase-resistant staining, contain intracellular Z-AAT polymers, and show reduced secretion of AAT into extracellular medium [17]. Moreover, in comparison with Pi*MM, Pi*ZZ liver organoids express lower levels of albumin or apolipoprotein B, two hepatocyte markers reported to be reduced in Pi*ZZ AATD liver patients [12,17]. In this study we further confirm that ZZ-ORG, and to a lesser extend MZ-ORG, are positive for PAS staining and accumulate Z-AAT polymers in hepatocytes.

Recent clinical studies provide clear evidence that patients with Pi*ZZ AATD-related liver disease often develop steatosis [12,22]. Hepatic steatosis is defined by the formation of cytosolic LDs in more than 5% of the hepatocytes and may reflect early liver injury [23] related to various pathologies, including non-alcoholic fatty liver disease (NAFLD) [24].

Our results show that liver ZZ-ORG contain more LD-positive cells and that LDs are larger than in MZ-ORG and, especially, than in MM-ORG. Likewise, according to lipidomic analysis, total lipid content is higher in ZZ-ORG, and also in MZ-ORG, relative to MM-ORG. These results support previously reported findings in Z-AAT transgenic mice models, and in cohorts of Pi*ZZ liver patients [12,22]. For example, liver steatosis was observed in Z-AAT overexpressing mice as well as in Pi*ZZ patients, as assessed by controlled attenuation parameter (CAP) [12]. Furthermore, it has been reported that liver patients with Pi*ZZ AATD have lower serum levels of triglycerides and very low-density lipoprotein cholesterol (VLDL), which may reflect an impaired hepatic secretion of lipids [12]. In fact, most of the lipid species evaluated in liver organoids were strongly augmented in ZZ-ORG compared to MM-ORG. Despite the net increase in many lipid species in ZZ-ORG, triacylglycerol (TAG) and cholesterol esters (CEs) together with ceramides (CERs) and cardiolipins (CLs) were most significantly elevated. TAG and CE are typically stored in LDs, and act as energy storages and protectors against deleterious effects of free fatty acids [25]. Moreover, TAG and CE are in the core of lipoproteins, which distribute lipids to the different tissues and organs [26]. The observed significant increment of TAG and CE might be related to higher numbers and sizes of intracellular LDs in ZZ- and in MZ-ORG in comparison to MM-ORG. On the other hand, the significantly lower percentage of phosphatidylcholine (PC) in ZZ-ORG than in MZ- or MM-ORG may also be related to the larger LDs in ZZ-ORG. The PC localizes on the surface of LDs and is described as an inhibitor of LDs’ enlargement [27]. These findings enable us to speculate that high number of large intrahepatic LDs may be related to hindered lipoprotein export from Pi*ZZ hepatocytes.

The levels of ceramides are also higher in ZZ-ORG relative to MM-ORG. Ceramides are precursors of sphingolipids and constituents of the cell membrane subdomains so called lipid rafts [28]. Ceramides increase upon cellular stimulation, and according to model membrane studies, an increase in ceramide/cholesterol content promotes the miscibility of lipid rafts [29]. This structural alteration in lipid rafts has been related to impaired liver function [30]. Finally, cardiolipins, which are also higher in ZZ-ORG, are among the most abundant lipids in the inner mitochondrial membrane, playing roles in mitochondria stability, metabolism, and dynamics [31]. It is conceivable that abnormalities in the content and/or the composition of cardiolipins may negatively impact mitochondrial function, with implications in diseases such as NAFLD [32].

Likewise, differential gene expression analysis between ZZ-ORG and MM-ORG revealed a divergent expression of genes encoding proteins related to lipid and fatty acids metabolism. For instance, in comparison with MM-ORG, ZZ-ORG showed a higher expression of *CD36*, *a* gene coding a transmembrane protein in charge of hepatic fatty acid uptake [33], which could contribute to the increased lipid content of hepatocytes. On the other hand, ZZ-ORG showed a lower expression of *FABP1*, a gene encoding a fatty acid binding protein responsible for the intracellular transport of long chain fatty acids and the assembly and export of lipoproteins. This hepatic protein might also play a role in controlling oxidative stress because its main function is to direct fatty acids to oxidation into the mitochondria [34]. Data from experimental models show that *FABP1* silencing results in intrahepatic TAG accumulation and the development of liver disease [35], while FABP1 can also act as a transcription factor activating genes contributing to TAG accumulation [36].

The liver is a central organ that maintains de novo lipogenesis and triglyceride secretion in a form of lipoproteins [37,38]. As mentioned above, patients with Pi*ZZ AATD-related liver disease have lower levels of circulating triglycerides and VLDL than non-AATD liver patients [12]. We found that the expression of the *APOB* gene is significantly lower in ZZ-ORG compared to MM-ORG [17]. The APOB protein provides stability for the lipoproteins, and the isoform APOB100 is a key component of HDL, LDL, and VLDL [39]. Apolipoproteins assemble in the ER, and thus TAG accumulation can induce ER stress and the inhibition of APOB synthesis [40]. The lower expression of APOB could contribute to the steatosis in Pi*ZZ AATD patients, as it has been similarly described for APOA1 and APOF in NAFLD [41,42]. Hence, altered lipoprotein transport and secretion, mirrored by the reduced expression of *APOB* and/or *FABP1,* and increased expression of *CD36* may, at least in part, explain the accumulation of lipids in Pi*ZZ liver. Concomitantly, other altered genes in ZZ-ORG also point to defective lipid processes such as fatty acid metabolism and bile acids secretion. ZZ-ORG showed overexpression of patatin-like phospholipase domain containing 8 (*PNPLA8*), a mitochondria membrane-bound phospholipase which cleaves phospholipid releasing fatty acids regulating membrane physical properties. The overexpression of *PNPLA8* is thought to cause mitochondrial abnormalities and dysfunction [43]. The *CROT* is another overexpressed gene found in ZZ-ORG that is involved in transport of medium- and long- chain acyl-CoA molecules out of the peroxisome to the cytosol and mitochondria. Other upregulated genes in ZZ-ORG, including several UDP glucuronosyltransferases (*UGT1A4*, *UGT1A5*, *UGT2B28*, *UGT2B10*) and ATP Binding Cassette Subfamily G Member 5, *ABCG5*, pointed to putatively altered bile secretion.

Likewise, GSEA functional readouts of the DEGs found in ZZ-ORG pointed to metabolic alterations, specifically to a downregulation of replication and transcription related genes, which are a hallmark of basal cellular activity, and a significant upregulation in genes of mitochondrial function, oxidative phosphorylation, and cholesterol biosynthesis, together with peroxisome and peroxisome lipid metabolism. In previous studies based on cell models, liver tissues of Pi*ZZ patients and transgenic mice expressing human Z-AAT described alterations in mitochondrial autophagy [44]. It is proposed that mitochondrial damage, including mitochondrial depolarization, increased permeability, and the activation of oxidative signaling pathways, occurs due to the intracellular accumulation of Z-AAT and lipids. In support of this idea, the alteration in the redox state of the liver ER was demonstrated in a transgenic Z-AAT expressing mice model [45]. In line, multi-omics analysis of MZ and ZZ edited iPSCs differentiated to hepatocytes (iHeps) revealed altered ER and mitochondrial morphology, reduced mitochondrial respiration, and branch-specific activation of the UPR [46]. Our results, both at transcription level and lipid alterations, also highlight the mitochondria as a player in AATD-related liver disease development. The DEG analysis revealed a significant number of mitochondrial genes of oxidative phosphorylation to be overexpressed in ZZ-ORG. As already mentioned, cardiolipins specifically were found to be higher expressed in ZZ-ORG than in MM-ORG [31].

Mitochondrial and peroxisomal proteins have functional overlap in lipid catabolism [47]. Among overexpressed DEGs in ZZ-ORG, we found peroxisome, peroxisome lipid metabolism, and cholesterol biosynthesis genes. Peroxisomes are also involved in lipid degradation and in the synthesis of bile acids [48]. Hence, increased peroxisomal β-oxidation can inhibit lipid hydrolysis and induce hepatic steatosis [49].

Bringing it all together, current knowledge suggests that AATD-associated lipid accumulation in hepatocytes can be initiated concomitantly with an increase in Z-AAT polymers and alterations in metabolic organelles, namely the ER, mitochondria, and peroxisome. Historical studies observed the increased number of peroxisomes located near dilated ER in Pi*ZZ hepatocytes, but no further experiments have been performed [50]. The peroxisomal involvement in AATD liver disease warrants further studies; therefore, patient-derived organoids are useful models for this purpose. Furthermore, patient-derived ZZ-, MZ-, and MM-ORG are appropriate models resembling the heterogeneity in degree of steatosis and liver damage found in patients with genetic variants of AAT. However, organoids are usually derived from a limited number of samples and conclusions obtained from comparison among genotype categories might not be representative for all patients. However, organoid models are interesting in revealing inter-individual differences and are useful models in the implementation of personalized medicine.

## 4. Materials and Methods

### 4.1. Culture of Human Liver Organoids

Liver organoids from patients with Pi*MZ AATD (who underwent cholecystectomy) and Pi*ZZ AATD (with hepatic failure that had liver transplant) and Pi*MM used as controls (unaffected tissue areas from a person undergoing surgical resection due to hepatocellular carcinoma) (Table 4) were developed as previously described [17]. All subjects signed the informed consent for the study and the research was approved by the ethics committee of Instituto de Salud Carlos III, Madrid, Spain.

Undifferentiated organoids were cultured in extracellular matrix BME-2 and expansion medium (EM) [51]. The differentiation into the hepatocytes was achieved by organoid culture in the differentiation medium (DM) for 15 days, as previously described [51].

To detect intracellular lipids, cytospins of differentiated organoids were prepared. Organoids were disaggregated, incubated in TrypLE Express (GibcoTM, ThermoFisher Scientific) at 37 °C, and centrifuged. After washing with phosphate-buffered saline (PBS), cells were spread on slides and fixed with 4% PFA.

### 4.2. Culture and Transfection of HepG2 Cell Line

The human hepatocellular carcinoma HepG2 cell line (ATCC No. HB-8065), obtained from the American Type Culture Collection, was grown in DMEM (Gibco^TM^, ThermoFisher Scientific, Waltham, MA, USA) supplemented with 10% FCS (Gibco^TM^, ThermoFisher Scientific, Waltham, MA, USA) and antibiotics (pen/strep) at 37 °C with 5% CO_2_. HepG2 cells were transiently transfected with the expression plasmid pCMV6 (Origene, Maryland, EE. UU), containing either the wild-type M allele or the mutated Z allele [52], with Lipofectamine^TM^ 2000 (Thermo Fisher Scientific, Waltham, MA, USA) in serum-free Opti-MEM culture medium (Gibco^TM^, ThermoFisher Scientific, Waltham, MA, USA) following the manufacturer’s recommendations. Then, transfection Opti-MEM was replaced by complete DMEM and cells were harvested after 48 h for subsequent experiments. HepG2 cells were washed with PBS and fixed with 4% paraformaldehyde (PFA) for further lipid staining.

### 4.3. Lipid Extraction for Mass Spectrometry Lipidomics

Lipids were extracted using a two-step chloroform/methanol procedure [53]. After extraction, the organic phase was transferred to an infusion plate and dried in a speed vacuum concentrator. The dry extract was re-suspended in 7.5 mM ammonium formiate in chloroform/methanol/propanol (1:2:4, *v*:*v*:*v*). All liquid handling steps were performed using Hamilton Robotics STARlet robotic platform with the Anti Droplet Control feature for organic solvent pipetting.

### 4.4. Mass Spectrometry Data Acquisition, Analysis, and Post-Processing

Samples were analyzed by direct infusion on a QExactive mass spectrometer (Thermo Scientific) equipped with a TriVersa NanoMate ion source (Advion Biosciences, New York, NY, USA). Samples were analyzed in both positive and negative ion modes with a resolution of Rm/z = 200 = 280,000 for MS and Rm/z = 200 = 17,500 for MSMS experiments, in a single acquisition. MSMS was triggered by an inclusion list encompassing corresponding MS mass ranges scanned in 1 Da increments. Both MS and MSMS data were combined to monitor CE, Chol, DAG, and TAG ions as ammonium adducts; LPC, LPC O-, PC, and PC O- as formiate adducts; and CL, LPS, PA, PE, PE O-, PG, PI, and PS as deprotonated anions. MS was only used to monitor LPA, LPE, LPE O-, LPG, and LPI as deprotonated anions; Cer, HexCer, and SM as formiate adducts; and cholesterol as ammonium adduct of an acetylated derivative.

Data were analyzed with in-house developed lipid identification software based on LipidXplorer. Data post-processing and normalization were performed using an in-house developed data management system. Only lipid identifications with a signal-to-noise ratio >5 and a signal intensity 5-fold higher than in the corresponding blank samples were considered for further data analysis.

### 4.5. Determination of Intracellular Lipid Content

Intracellular accumulation of lipids was determined by using Oil-Red-O (ORO) (Sigma Aldrich, Madrid, Spain) which binds to neutral lipids. Before staining, preparations were washed once with PBS followed by 60% isopropanol. Staining was carried out at 37 °C for 5 min, then washing was performed with 60% isopropanol and PBS, and finally preparations were visualized using Leica DFC 7000T microscope (Leica Microsystems, Wetzlar, Germany). For quantification, ORO staining was extracted with 1 mL 100% isopropanol and the optical density of the samples was measured at a wavelength of 510 nm using a microplate reader Infinite 200 PRO spectrophotometer (Tecan, Männedorf, Switzerland). Cells were also stained with crystal violet solution and dye was extracted for quantification with 50% 0.1 M sodium citrate (Sigma Aldrich, Madrid, Spain) and 50% ethanol and absorbance was measured at 590 nm. Alternatively, organoids-derived cells were stained with hematoxylin, washed twice with distilled water, and, finally, visualized using Leica DFC 7000T microscope (Leica Microsystems). For quantification, randomly selected fields were captured and lipid content was quantified using IP Win32 software v4.5.0.29.

### 4.6. Western-Blot Analysis of AAT

Native and polymeric forms of AAT were detected in HepG2 cells transfected with M and Z alleles as previously described [17]. Quantification of the Western blot bands of the AAT protein in MM-ORG and ZZ-ORG was performed with Image J software v1.48. The antibodies used for Western blotting were: rabbit polyclonal antisera against human AAT (1:1000; Dako, Denmark); antibody D11 (1:800) [54] for the detection of AAT aggregates; and monoclonal antibody AC-74 anti-beta-actin (1:5000; Sigma Aldrich). The secondary antibodies used were chicken anti-mouse IgG-HRP (sc-2954 Santa Cruz Biotechnology, USA) and donkey anti-rabbit (Cytiva Europe GMBH, Barcelona Spain), both at 1:5000 dilution.

### 4.7. Detection of Intracellular AAT by Immunofluoresce

Immunostaining was performed on HepG2 cells transfected with M and Z alleles cultured on coverslips that were fixed at room temperature for 10 min with PBS containing 2% paraformaldehyde (PFA) followed by another 10 min with PBS 4% PFA. After several washes with PBS, the cells were permeabilized in PBS containing 0.1% Triton X-100 and 1% BSA (PBS-TS). The cells were then incubated overnight at 4 °C with the primary antibody against AAT (1:250; Dako, Denmark) diluted in PBS-TS, and after washing with PBS they were incubated for 1 h at room temperature in PBS-TS with ant-rabbit Alexa-488-conjugated secondary antibody (1:400; Invitrogen). Following extensive washes, the preparations were stained with 4′-6-diamidine-2-phenylindole (DAPI; 1:5000; Sigma-Aldrich) and after washing, the cells were immediately mounted with Fluoromount g (Southern Biotech, Birmingham, AL, USA). The preparations were examined under a fluorescence microscope Zeiss Ax10 (Zeiss, Oberkochen, Germany).

### 4.8. Transcriptomic Analysis of Liver Organoids (RNA-Seq)

Total RNA was isolated from organoid cells using TriReagent (Sigma Aldrich, Madrid, Spain) followed by a DNaseI digestion step to ensure that the samples were not contaminated with genomic DNA. The purity of RNA was assessed using Agilent RNA 6000 Nano Kit and the Agilent 2100 Bioanalyzer. TruSeq Stranded mRNA Kit (Illumina) was used for library preparation based on the recommendations from the manufacturer. The sequencing was performed at the Genomics Unit (ISCIII) on a NextSeq 500 sequencer using 75 base read lengths in paired-end mode. RNA-Seq data were analyzed by the Bioinformatics Facility (ISCIII) with an RNA-seq pipeline (https://github.com/BU-ISCIII/rnaseq-nf) written in Nextflow (https://www.nextflow.io/) based on the those written by nf-core (https://nf-co.re/) (https://github.com/nf-core/rnaseq) (accessed on 21 December 2021). (Accession no. GSE220537).

Functional characterization was investigated using the STRING database (https://string-db.org/) (accessed on 23 May 2022) to find out functional enrichments within the set of differentially expressed genes (DEGs) [55].

For GSEA Preranked, genes were pre-ranked according to the statistic test of fold change in ZZ-ORG versus MM-ORG obtained in the RNA-Seq analysis, setting ‘gene set’ as the permutation method and with 1000 permutations. Clustering of enriched GO terms at *p*-value < 0.01 was analyzed using EnrichmentMap for Cytoscape. An unsupervised hierarchical cluster heatmap was plotted using Morpheus (https://software.broadinstitute.org/morpheus) (accessed 23 May 2022) with the expression of genes in the leading edge of the significantly enriched gene sets from the Molecular Signature Database (MSigDB) KEEG_Oxidative phosphorylation, REACTOME_Peroximal Lipid metabolism, REACTOME_Cholesterol biosynthesis, WP_Mitochondrial complex IV assembly, and KEGG_Peroxisome.

### 4.9. Data Processing and Statistical Analysis

Graphical data presentation was performed using GraphPad Prism [Version 9.1.2 (226), Dotamatics]. Student’s t-test was applied to compare two sample means on one variable. The normally distributed data presented as a mean and standard deviation of the mean (SD). If the normality test failed, the nonparametric Kruskal–Wallis one-way analysis followed by Mann–Whitney rank-sum test was performed. A *p*-value below 0.05 considered as a significant.

## Figures and Tables

**Table 1:** Total lipid amount measured in organoids with the different genotypes.

Class ^1^		MM		MZ				ZZ			
		Media (pmol)	SD *	Media (pmol)	SD	FC **	*p*-Value ***	Media (pmol)	SD	FC	*p*-Value ***
Cholesterol	Chol	5070.61	488.56	5776.7	357.55	1.14	0.0352	5793.16	678.17	1.14	0.1075
Phosphatidylcholine	PC	3814.2	391.59	4848.09	426.64	1.27	0.0746	4116.96	398.06	1.08	0.3082
Phosphatidylethanolamine	PE	2116.96	207.09	2001.29	144.14	0.95	0.3366	2257.92	284.74	1.07	0.3556
Phosphatidylserine	PS	1076.2	145.32	1025.35	95.45	0.95	0.3933	1071.83	101.36	1	0.4908
Triacylglycerol	TAG	1065.28	60.73	3325.34	312.73	3.12	0.0079	3112.48	399.46	2.92	0.0168
Phosphatidylinositol	PI	525.25	26.77	775.52	41.67	1.48	0.0054	601.58	53.74	1.15	0.1475
Phosphatidylethanolamine (-ether)	PE-O	460.33	43.84	641.14	49.71	1.39	0.0267	868.37	108.36	1.89	0.0243
Diacylglycerol	DAG	376.61	25.09	417.04	39.62	1.11	0.2227	466.18	65.62	1.24	0.1527
Sphingomyelin	SM	290.89	31.92	350.47	22.77	1.2	0.1053	367.73	43.07	1.26	0.1154
Ceramide	Cer	231.32	18.2	363.72	18.65	1.57	0.0035	434.46	57.11	1.88	0.0297
Hexosylceramide	HexCer	87.76	9	41.95	1.72	0.48	0.0163	65.46	6.45	0.75	0.0608
Phosphatidate	PA	69.81	5.12	76.21	5.81	1.09	0.2279	83.79	10.23	1.2	0.1552
Phosphatidylglycerol	PG	47.58	4.89	60.24	4.36	1.27	0.0631	81.52	10.36	1.71	0.0317
lyso-Phasphatidylethanolamine	LPE	25.94	2.46	16.82	0.57	0.65	0.0295	44.62	5.14	1.72	0.0247
Cardiolipins	CL	25.06	1.6	54	2.69	2.15	0.001	47.67	7.27	1.9	0.0417
Phosphatidylcholine (-ether)	PC-O	24.71	2.34	50.62	4.23	2.05	0.0058	59.8	8.12	2.42	0.0204
lyso-Phasphatidylcholine	LPC	22.38	2.17	17.96	2.68	0.8	0.1359	49.03	4.96	2.19	0.0099
Cholesterol esters	CE	13.47	0.59	120.21	7.92	8.92	0.0026	173.25	24.02	12.86	0.0109
lyso-Phasphatidate	LPA	4.61	0.24	0	0	0	0.0014	7.48	1.04	1.62	0.0519
lyso-Phosphatidylserine	LPS	3.12	0.53	0.71	0.71	0.23	0.0287	4.28	0.49	1.37	0.092
lyso-Phosphatidylinositol	LPI	2.7	0.41	1.51	1.51	0.56	0.2583	2.54	0.32	0.94	0.3845
lyso-Phosphatidylethanolamine (-ether)	LPE-O	2.34	0.22	0	0	0	0.0043	6.6	0.72	2.81	0.01
lyso-Phosphatidylglycerol	LPG	1.59	0.1	0	0	0	0.0019	3.66	0.5	2.31	0.0246
lyso-Phosphatidylcholine (-ether)	LPC-O	0.86	0.18	0	0	0	0.0209	3.04	0.28	3.53	0.0025

^1^ Lipid species are ordered from most abundant to less abundant in MM-ORG, divided in the three groups of High abundant (>1000 pmol), Intermediate abundant (10 to 1000 pmol), and Low abundant (<10 pmol). * SD: Standard deviation; ** FC: Fold change; *** *p*-value is referred to in comparison with MM-ORG.

**Table 2:** Percentage of the lipid species representing the relative abundance within each genotype (molar fraction).

Class		MM		MZ			ZZ			
		Media (%)	SD *	Media (%)	SD *	*p*-Value MM	Media (%)	SD *	*p*-Value MM	*p*-Value MZ
Cholesterol	Chol	33	0.0801	28.99	0.5554	0.017	29.35	0.0865	6.87 × 10^-6^	0.5837
Phosphatidylcholine	PC	24.8	0.4197	24.24	0.4711	0.4215	20.95	0.41	0.0028	0.0066
Phosphatidylethanolamine	PE	13.78	0.1345	10.03	0.1567	0.0001	11.42	0.1663	0.0005	0.0005
Triacylglycerol	TAG	6.98	0.2678	16.61	0.3737	0.0001	15.74	0.3171	3.70 × 10^-5^	0.1512
Phosphatidylserine	PS	6.96	0.2644	5.14	0.288	0.0098	5.46	0.2001	0.0126	0.4155
Phosphatidylinositol	PI	3.45	0.1424	3.9	0.087	0.0663	3.07	0.0792	0.0991	0.0022
Phosphatidylethanolamine (-ether)	PE-O	3	0.042	3.21	0.0287	0.0179	4.39	0.0482	3.01 × 10^-5^	0.0001
Diacylglycerol	DAG	2.47	0.0944	2.08	0.0472	0.037	2.35	0.074	0.3865	0.0479
Sphingomyelin	SM	1.89	0.0695	1.76	0.0171	0.1905	1.87	0.0507	0.7852	0.1581
Ceramide	Cer	1.51	0.0316	1.83	0.0668	0.0254	2.19	0.0876	0.0093	0.0328
Hexosylceramide	HexCer	0.57	0.0343	0.21	0.0071	0.007	0.33	0.0062	0.0174	0.0002
Phasphatidate	PA	0.46	0.0108	0.38	0.0072	0.007	0.42	0.0037	0.0843	0.0135
Phosphatidylglycerol	PG	0.31	0.0044	0.3	0.0073	0.4399	0.41	0.0057	0.0002	0.0004
lyso-Phosphatidylethanolamine	LPE	0.17	0.008	0.08	0.0035	0.0035	0.23	0.0048	0.0068	0
Cardiolipins	CL	0.16	0.0052	0.27	0.0079	0.0007	0.24	0.0117	0.0121	0.0946
Phosphatidylcholine (-ether)	PC-O	0.16	0.0031	0.26	0.0264	0.0675	0.3	0.0098	0.0025	0.2206
lyso-Phosphatidylcholine	LPC	0.15	0.0022	0.09	0.0068	0.0091	0.25	0.0033	4.18 × 10^-5^	0.0003
Cholesterol esters	CE	0.09	0.0103	0.6	0.0103	3.88 × 10^-6^	0.87	0.022	0.0001	0.002
lyso-Phosphatidate	LPA	0.03	0.0032	0	0	0.0106	0.04	0.0028	0.1618	0.0055
lyso-Phosphatidylserine	LPS	0.02	0.0039	0	0.004	0.0425	0.02	0.0002	0.7866	0.0483
lyso-Phosphatidylinositol	LPI	0.02	0.001	0.01	0.0067	0.2457	0.01	0.0011	0.0409	0.4465
lyso-Phosphatidylethanolamine (-ether)	LPE-O	0.02	0.0011	0	0	0.0052	0.03	0.0004	0.0014	0.0014
lyso-Phosphatidylglycerol	LPG	0.01	0.0005	0	0	0.0023	0.02	0.0007	0.0009	0.0013
lyso-Phosphatidylcholine (-ether)	LPC-O	0.01	0.0007	0	0	0.017	0.02	0.0015	0.01	0.0091

* SD: Standard deviation.

**Table 3:** Functional annotations of differentially expressed genes in ZZ-ORG versus MM-ORG.

Cluster	Term ID	Term Description	Observed Gene Count	Background Gene Count	Strength	FDR *
(1)	GO:0003779	Actin binding	15	438	0.93	8.25 × 10^−7^
Actin Cytoskeleton	GO:0007010	Cytoskeleton organization	18	1126	0.6	0.0017
	GO:0031982	Vesicle	29	3879	0.27	0.0393
(2)	GO:0019222	Regulation of metabolic process	62	6948	0.32	1.45 × 10^−9^
Regulation of metabolic processes	GO:0060255	Regulation of macromolecule metabolic process	60	6407	0.34	1.45 × 10^−9^
	GO:0010468	Regulation of gene expression	51	4813	0.4	3.64 × 10^−9^
	GO:0006950	Response to stress	37	3485	0.4	4.68 × 10^−6^
	GO:0030162	Regulation of proteolysis	15	747	0.68	8.76 × 10^−5^
	GO:0001817	Regulation of cytokine production	16	742	0.71	1.82 × 10^−5^
	GO:0042981	Regulation of apoptotic process	21	1550	0.5	0.00019
	GO:0006954	Inflammatory response	11	515	0.7	0.0011
	GO:0006955	Immune response	18	1588	0.43	0.0061
(3)	GO:0032787	Monocarboxylic acid metabolic process	13	515	0.73	0.0119
Fatty acid metabolic processes	GO:0019752	Carboxylic acid metabolic process	16	853	0.61	0.0138
	GO:0006631	Fatty acid metabolic process	9	311	0.79	0.0448
	KEGG-hsa00982	Drug metabolism - cytochrome P450	7	64	1.37	4.22 × 10^−6^
	KEGG-hsa00140	Steroid hormone biosynthesis	5	59	1.26	0.00056
	KEGG-hsa04976	Bile secretion	5	89	1.08	0.0025
(4)	GO:0030054	Cell junction	28	2075	0.43	0.0011
Plasma membrane	GO:0031226	Intrinsic component of plasma membrane	22	1703	0.42	0.0229
(5)	GO:0031090	Organelle membrane	27	3548	0.39	0.002
Endomembrane system	GO:0005794	Golgi apparatus	17	1584	0.54	0.0027
	GO:0034703	Cation channel complex	7	214	1.02	0.0027
	GO:0012505	Endomembrane system	30	4542	0.33	0.003
	GO:0098797	Plasma membrane protein complex	9	547	0.72	0.0079
	GO:0008194	UDP-glycosyltransferase activity	6	143	1.13	0.0092

* FDR: False Discovery Rate.

**Table 4:** Clinical features of patients included in deriving hepatic organoid lines.

Organoids	Sex	Age	Reason of Surgery	Serum AAT	GPT	GOT	GGT	Platelets	Glucose	Albumin	Bilirubin
Genotype		(Years)		(g/L)	(U/L)	(U/L)	(U/L)	(×1000/uL)	(mg/dL)	(g/dL)	(mg/dL)
					(5–45) *	(5–33) *	(8–61) *	(140–450) *	(70–110) *	(3.5–5) *	(0.2–1) *
MM	F	78	Hepatocellular carcinoma	ND	31	31	49	246	113	4.5	0.3
MZ	M	82	Cholecystectomy	ND	64	36	33	126	110	3.9	0.3
ZZ	M	1	Hepatic failure. Liver transplant	0.41	199	360	551	92	71	3	3.67

* Range of normal values.; ND: non-determined.

## Data Availability

The data presented in this study are openly available in the Gene Expression Omnibus (GEO) repository, reference number (GSE220537).
